# Heterogeneous response to target therapy in metastatic papillary renal cell carcinoma evaluated by morphologic and metabolic multimodality imaging: A case report

**DOI:** 10.1097/MD.0000000000018093

**Published:** 2019-12-16

**Authors:** Emanuele Naglieri, Artor Niccoli Asabella, Anna Giulia Nappi, Claudia Carella, Cristina Ferrari, Giuseppe Rubini

**Affiliations:** a Medical Oncology Unit, IRCCS Istituto Tumori “Giovanni Paolo II”; b Nuclear Medicine Unit, Department of Interdisciplinary Medicine, University of Bari “Aldo Moro”, Bari, Italy.

**Keywords:** 18F-FDG PET/CT, heterogeneous response, multimodality imaging, papillary renal cell carcinoma, target therapy

## Abstract

**Rationale:**

Papillary renal cell carcinoma (PRCC) accounts for about 15% to 20% of renal cell carcinoma and is histologically distinguished in type I and type II. The last one is associated with poorer prognosis.

Treatment options for PRCC patients are surgery, immunotherapy, revolutionized by Nivolumab, and other target-therapy with an improvement in overall survival. Heterogenous response and a pseudo-progression may be observed in the initial phase of biological treatment that could induce premature discontinuation.

**Patient concerns:**

We present the case of a 44-year-old woman with left cervical palpable mass increased in size and without concomitant disease or previous surgery.

**Diagnosis:**

Neck ultrasonography, contrast-enhanced Computed Tomography, and 18F-FDG PET/CT were performed with the detection of lymph nodes involvement and a left renal lesion.

**Interventions:**

The patients underwent left radical nephrectomy and homolateral cervical and para-aortic lymphadenectomy, with histological diagnosis of PRCC, type II. After disease relapse, the inter-aortocaval lymph node was laparoscopically removed. Following the detection of further disease relapse in several lymph nodes and the lung, several lines of target-therapy were started; then disease progression and worsening of clinical and hematological status led us to start Nivolumab as last-line therapy.

**Outcomes:**

A heterogeneous response to therapies was documented with morphological and nuclear medicine imaging, however the concomitant deterioration of performance status and liver function led to discontinuation of Nivolumab; then the patient died, 30 months after diagnosis.

**Lessons:**

Here we describe the clinical case and radiological and nuclear medicine imaging investigations performed by our patient, highlighting that 18F-FDG PET/CT shows greater adequacy in assessing the response to therapy, avoiding premature drug discontinuation, and ensuring better management of a patient with advanced PRCC.

## 1 Introduction

Papillary renal cell carcinoma (PRCC) accounts for about 15% to 20% of renal cell carcinoma (RCC) representing the second most common histological type after clear cell variant.^[1]^

The peculiarity of this histotype is the presence of tubule-papillary architecture that, about cytological features, allows a further division in type I and type II, with different prognostic outcome.^[2]^ Type I and type II PRCC present a different biological background: type I is associated with MET-proto-oncogene alterations, while type II is associated with CDKN2A, SETD2, and TFE3 mutations; type 2 PRCC correlates with poor survival.^[3]^

Treatment options for metastatic PRCC patients are the upfront nephrectomy with cytoreductive intent and different systemic therapies.^[4]^ The immunotherapy with the old interferon alfa (INFα) and Interleukin 2 (IL-2), characterized by poor clinical benefits and high toxicity, has been revolutionized by the synthesis of new anti-programmed death 1 (PD-1) monoclonal antibodies (nivolumab).^[5,6]^

Other therapies are tyrosine kinase inhibitors (TKI) targeting the vascular endothelial growth factor (VEGF) receptors and inhibitors of the mammalian target of rapamycin (mTOR). Sunitinib, pazopanib, temsirolimus, and bevacizumab (in combination with INFα) are approved in the first-line setting, whereas sorafenib, cabozantinib, axitinib, and everolimus (as a single agent or in combination with lenvatinib) are approved as second-line agents.

The new biological, anti-angiogenic, or immunological drugs, have an acceptable safety profile and could improve overall survival, but the heterogeneous response could induce premature discontinuation.^[7,8,9]^ Can metabolic and morphologic multimodality imaging help oncologists to assess the correct response to therapy?

## 2 Case report

**Figure 1:**
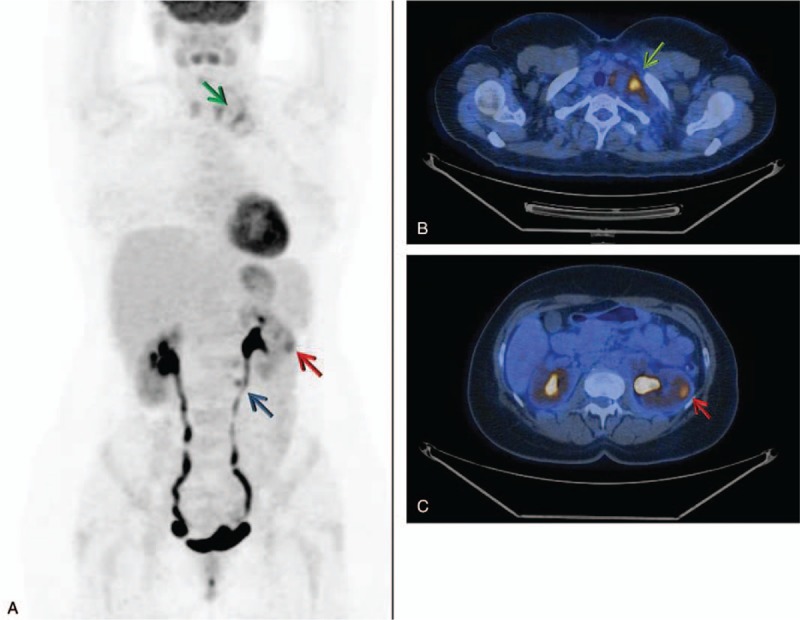
18F-FDG PET/CT (A) MIP and (B-C) axial fusion images showed increased glucose metabolism in the left kidney (red arrows) and in several homolateral lymphnodes in cervical, supra-clavicular (green arrows), and para-aortic sites (blue arrow).

**Figure 2:**
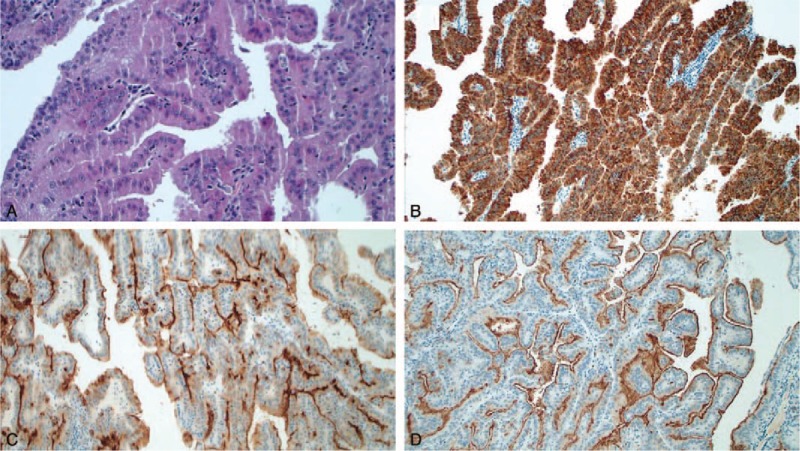
Histologic images of papillary renal cell carcinoma (PRCC) type 2 with predominant papillary pattern. Original magnification ×200 (A) Hematoxylin-eosin shows large cells with pseudo-stratified atypical nuclei and abundant cytoplasm characterized by eosinophilic and granular content. (B) Immunohistochemical image AMACR positive. (C) Immunohistochemical image CD10 positive. (D) Immunohistochemical image RCC positive.

A 44-year old female patient, in July 2015, came with a history of left cervical palpable mass which gradually increased in size in the last 3 weeks. She denied concomitant diseases or previous surgery. The hematological tests, including the renal function index and urinalysis, were normal except for the increase of the ESR (50 mm/h). Neck ultrasonography showed several lymph nodes of increased size (34 mm maximum diameter) in left cervical and homolateral supra-clavicular sites. The contrast-enhanced Computed Tomography (CT) confirmed the left cervical lymph-adenopathy and detected also para-aortic lymph node involvement and a hypodense lesion of 2.9 × 3.2 mm in the left kidney. Whole Body 18F-Fluorodeoxyglucose Positron Emission Tomography/Computed Tomography (18F-FDG PET/CT) confirmed the increased glucose metabolism in the left kidney (SUV max 6.5) and several homolateral lymph nodes in cervical, supraclavicular and para-aortic sites (Fig. 1). In August 2015 the patient underwent left radical nephrectomy and homolateral cervical and para-aortic lymphadenectomy. The histopathological examination of the specimen resulted in Papillary Renal Cell Carcinoma (type II, Fuhrman III). Immunohistochemical staining was positive for CKAE1/AE3, AMACR, and Vimentina. Ki67 was 55% and the disease stage was T1aN1M1 (Fig. 2). The postoperative period was uneventful.

**Figure 3:**
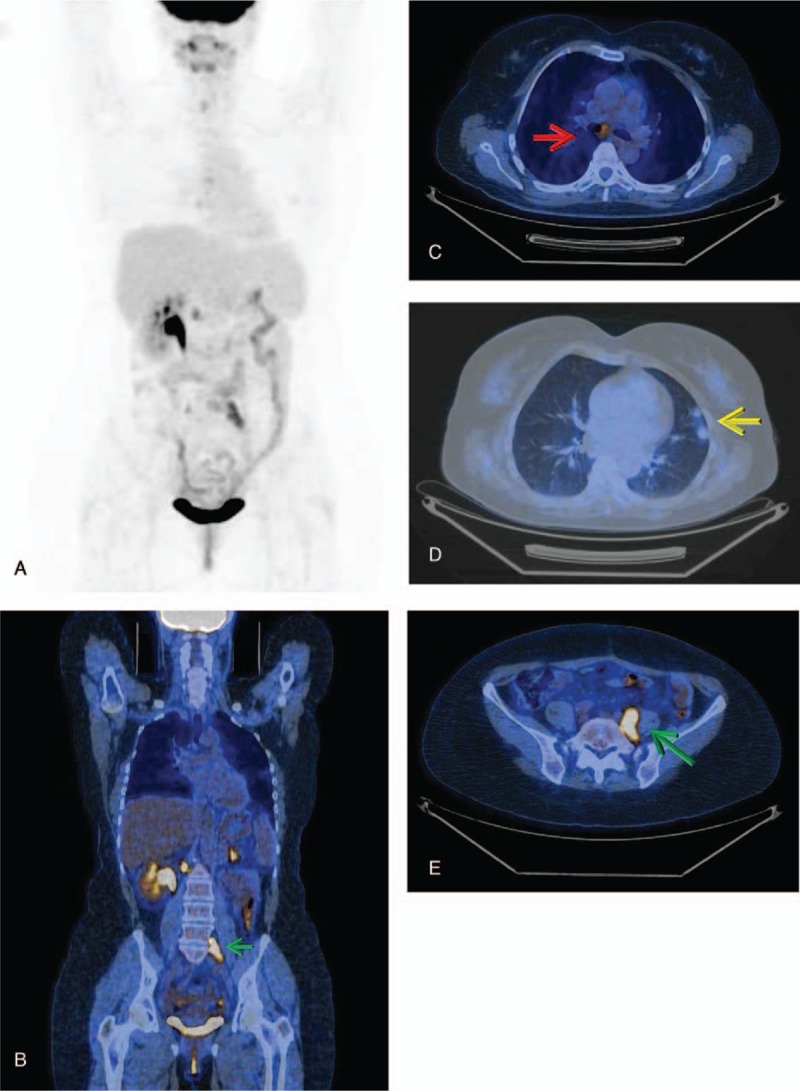
18F-FDG PET/CT (A) MIP, (B) coronal, and (C-D-E) axial fusion images detected disease progression with an increased uptake of radioactive tracer in several lymphnodes in subcarinal (red arrow) and para-aortic sites (green arrows). Two lung lesions (yellow arrow) showed an increased uptake of 18F-FDG. Left kidney is not visible because of previous left nephrectomy.

In October 2015 disease relapse was detected in an inter-aortocaval lymph node that was laparoscopically removed. Following the CT evidence of 2 suspected lung lesions (maximum diameter of 3 mm), Sunitinib 50 mg/day administration was started. In December 2015 lung lesions were unchanged, while a subcarinal hypodense lymph node of 13 × 23 mm and 2 left abdominal nodules of 13.5 mm of diameter were suspected for disease progression (Fig. 3).

**Figure 4:**
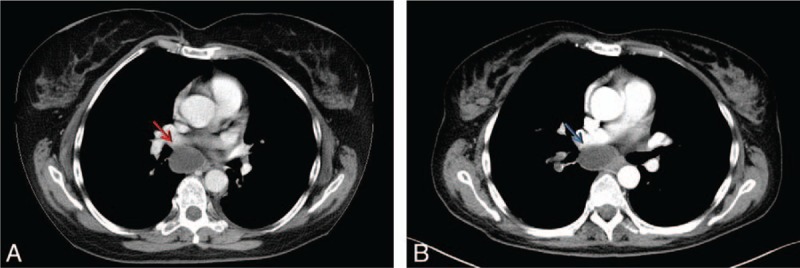
Enhanced-CT axial sections. (A) In 2016, the subcarinal lymphnode (red arrow) changed from hypodense to isodense and appeared reduced in size. (B) One year later, the same lesion (blue arrow) appear stable.

**Figure 5:**
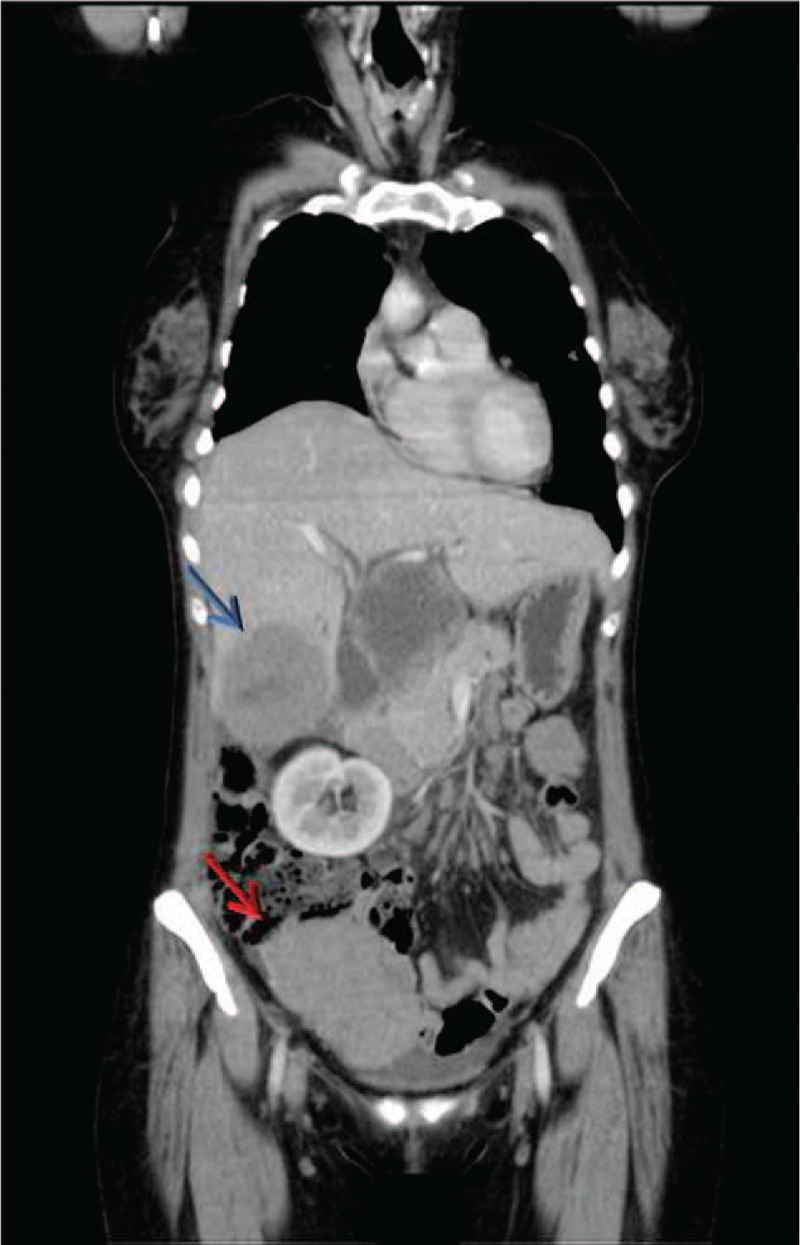
Enhanced-CT coronal section showed an increase in size of right abdominal retro-peritoneal lesion (red arrow) and peri-hepatic hilus lymphnodes (blue arrow) with necrotic central area and significant compression of the adjacent structures.

In February 2016 the CT scan showed a clear numerical and dimensional increase in lung lesions (with the biggest one of the maximum diameter of 11.4 mm), an enlargement of the subcarinal lymph node (30 × 53 mm) with a slight reduction in the diameter of the 2 abdominal lesions (10.4 mm). Axitinib 10 mg/day was started as second-line therapy. In July 2016 the radiologic assessment showed stable disease in abdominal findings; the pulmonary nodules although appearing slightly increased were less hypodense. The subcarinal lymph node also presented the same transformation from hypodense to isodense (Fig. 4) and was also smaller (25 × 30 mm). Concerning the patient's mixed response to therapy and good tolerability of the drug, the multidisciplinary team recommended to continue Axitinib at the increased dose of 7 mg/2/day for the following 3 months. In January 2017, after 10 months of Axitinib, the progression of disease status was evident. Bone metastatic involvement (metastasis of the V thoracic vertebrae) was documented. The laboratory panel showed anemia (HGB 10.1 g/dl), hepatic dysfunction (ALT 95 U/L, AST 67 U/L) and the patient reported abdominal pain, serotine fever well responsive to paracetamol 500 mg and codeine 30 mg. In February 2017 the Nivolumab immunotherapy 10 mg/die endovenous was started. In August 2017 the CT scan documented a mixed response with the stability of number and diameter of lung lesions and of subcarinal (Fig. 4) and para-aortic lymph nodes and with an increase in the size of abdominal retroperitoneal lesion (10.5 × 10 mm) and peri-hepatic hilus lymph nodes (5.4 × 6.1 mm) with necrotic central area. At the same time there was a deterioration in performance status (from ECOG 0–1 to ECOG 2–3), anemia (HGB 9.3 g/dl) and liver function index (ALT 186 U/L, AST 75 U/L, ALP 1500 U/L). Nivolumab was discontinued in November 2017 after the last CT scan documented further increase of the abdominal retroperitoneal lesions (50 × 51 mm) and peri-hepatic hilus lymph nodes (125 × 100 mm) with a necrotic central area and significant compression of the adjacent structures (Fig. 5). The patient died due to the disease in February 2018, 30 months after the diagnosis.

## 3 Discussion

The etiological factors of PRCC include hereditary, heavy metal exposure, tobacco, obesity, and drug abuse. The mean age of patient presentation is 61.8 years (range 22–83). PRCC often clinically appears with an abdominal mass, pain, and hematuria. In our patient, the first clinical sign was the left latero-cervical lymphadenopathy. The predominance of the papillary pattern is the main histological feature of PRCC. In type-II, papillae are lined by large cells with abundant eosinophilic cytoplasm and the nuclei show frequently pseudo stratification and prominent nucleoli. These are considered high-grade PRCC.^[10]^ Our patient was histologically type-II with nuclear grade III according to Fuhrman grading.

The comparison of the type I PRCCs with type II PRCCs revealed that the last one was significantly associated with a greater stage and grade of microvascular invasion. The overall and disease-free survival rate was 89% and 92% in type I tumors and 55% and 44% in type II tumors, respectively.^[2]^

The surgery is the treatment of choice in metastatic patients, with good performance status and low systemic tumor burden: the cytoreductive nephrectomy to remove the primary renal tumor and the metastasectomy to remove distant metastatic foci.^[4]^

Chemotherapy has low response rate and high toxicity, consequently, it has been replaced by targeted therapies: smart molecules that inhibit intracellular tyrosine kinase (TKIs) pathways with anti-angiogenic action against VEGF receptors and/or other molecular receptors (PDGFR), VEGF-directed monoclonal antibody and mammalian target of rapamycin (mTOR) inhibitory small molecules. Also, a retrospective analysis suggested a longer survival in patients treated with targeted therapies after cytoreductive nephrectomy.^[7,8,11,12]^

Despite the histological and molecular characteristics between non-clear cell renal cell carcinoma and clear cells (ccRCC) are different, the therapeutic approach is the same but different in efficacy.^[9]^ In fact, during treatment with anti-angiogenic drugs in the first line (eg sunitinib), progression-free survival (PFS) is between 9 to 12 months in patients with metastatic ccRCC, while it is between 1.6 and 6.6 months for metastatic PRCC.^[3]^

The overall survival (OS) of PRCC patients treated with targeted therapy is significantly longer than the OS of those not treated with targeted therapy (median 22.5 vs 6.3 months).^[13]^

Our patient treated with Sunitinib was shifted after 5 months to Axitinib; this drug was continued for 10 months until disease progression and unacceptable toxicity. Subsequently, she underwent Nivolumab administration as the last line of treatment.

Sunitinib has been approved as first-line agents for the treatment of advanced RCC; it is an oral multi-targeted inhibitor (TKI) with higher response rates and significant improvement in median PFS compared to patients receiving IFN-α and with a significantly superior overall response rate (ORR) compared to placebo subgroup. Regarding patients with PRCC, 3 randomized, phase 2 trials showed a longer progression-free survival in patients treated with sunitinib compared to everolimus.^[9]^

Axitinib has been approved for the second-line treatment of advanced RCC; it is an oral TKI with a statistically significant improvement in median PFS and ORR compared to Sorafenib in RCC patients with progressive disease after previous systemic therapy.^[14]^

Our patient showed stable disease on abdominal findings but an increase in size and a change in density in other metastatic foci (lung lesions and subcarinal lymph node).

Heterogeneous and mixed response to tyrosine kinase inhibitor was observed in patients with mRCC. This could link with intratumor heterogeneity (WITH), the molecular phenomenon that underlies the genetic complexity of mRCC.

This suggests the existence of different cellular sub-clones derived from a common progenitor with different differentiation and different drug resistance within regions of the same tumor and various metastases.

As a clinical result, metastatic lesions of the same patients might show heterogeneous progression patterns in radiologic imaging, due to the specific cellular sensitivity to the drug.

Currently, the RECIST criteria are widely used to determine the tumor response assessment, but may not be adequate to evaluate response to anti-angiogenic therapies even because size changes of lesions may be lag behind the actual response. Targeted therapy usually induces only mild lesion shrinkage and metastases can even increase in size while the drug is acting with efficacy.^[15,16]^

Indeed, our patient showed a slight increase in the size of the pulmonary lesions after Axitinib that than remained stable until the patient's exitus. Also, the subcarinal lymph node was Axitinib-sensitive showing size and density reduction.

Rather than morphological imaging methods, 18F-FGD PET/CT may be helpful to evaluate the biological tumor response^[17,18]^ and to decide the therapeutic choice, avoiding a premature cessation of efficacious treatment or the continue of a non-efficacious, toxic, and expensive one, improving clinical management of cancer patients treated with TKIs. 18F-FDG radiotracer uptake allows the rapid identification of non-responders who could benefit from alternative treatments; changes in glucose metabolism occurs surely before changes in tumor size.^[19,20]^

Following the failure of systemic treatment with targeted therapy probably due to the development of resistance mechanism and also to toxicity, Nivolumab was chosen for a third-line treatment and recently approved as an alternative sequential therapy for advanced mRCC.

Nivolumab is a humanized IgG4 antibody specific for binding the PD-1, preventing the interaction with its ligands PD-L1 and PD-L2. It reverses the tumor-induced immune suppression blocking the immune checkpoint and reactivating the antitumor immune response, also stimulating the immunologic memory, remarkably compared to other drugs. Nivolumab showed an acceptable safety profile, generating an effective immunologic memory and a longer OS.^[5,6]^

CheckMate 025 demonstrated the significant superiority of Nivolumab in ORR and OS in patients affected by mRCC pre-treated with anti-angiogenic therapy, compared to everolimus, with lower related adverse events and better quality of life.^[21]^

During the initial phase of immune-checkpoint inhibitory treatment, an increase in tumor size (possibly due to lymphocyte infiltration) and the appearance of new lesions may occur. This phenomenon is defined as pseudo-progression and has frequently led to drug discontinuation, especially in the late-line setting. Older age is the main risk factor associated with this rapid progression and the hyper-progressive showed a worse overall survival.^[22]^

Our patient showed a heterogeneous response to Nivolumab documented with CT scan: lung lesions, subcarinal and para-aortic lymph nodes remained the same in size and number, while there was an increase in size of abdominal retroperitoneal lesions (10.5 × 10 mm) and peri-hepatic hilus lymph nodes characterized of significative dimension (5.4 × 6.1 mm) with necrotic central area.

PRCC is an immune responsive tumor although we can suggest that several cellular subclones with different antigenic expression, can show different immunologic response. Nivolumab efficacy may develop in different ways among regions of the same tumor and different metastases, due to the intra-tumor heterogeneity phenomenon well known in the literature.

The OS of our patient was 30 months, a little longer than expected, with an important deterioration in performance status only in the last 2.5 months.

## 4 Conclusions

PRCC Type-II appears to have a poorer prognosis than ccRCC, so early diagnosis is important for better clinical management. The introduction of new biological, anti-angiogenic, or immunological drugs could lead to survival improvement.

Heterogeneous response to different therapies and a pseudo-progression in the initial phase of biological treatment may be observed but this does not necessarily reflect therapeutic failure and does not have to lead us to premature drug discontinuation.

Rather than morphological imaging methods based on RECIST, 18F-FDG PET/CT recently showed greater adequacy in assessing the response to therapy by ensuring better management of the patient with advanced PRCC.

## Author contributions

**Conceptualization:** Emanuele Naglieri, Artor Niccoli Asabella, Claudia Carella.

**Investigation:** Emanuele Naglieri, Artor Niccoli Asabella, Cristina Ferrari.

**Supervision:** Claudia Carella, Giuseppe Rubini.

**Writing – original draft:** Emanuele Naglieri, Artor Niccoli Asabella, Anna Giulia Nappi.

**Writing – review & editing:** Anna Giulia Nappi, Cristina Ferrari, Giuseppe Rubini.
